# Enhanced effects of aminolaevulinic acid-based photodynamic therapy through local hyperthermia in rat tumours

**DOI:** 10.1038/sj.bjc.6601036

**Published:** 2003-07-15

**Authors:** D K Kelleher, J Bastian, O Thews, P Vaupel

**Affiliations:** 1 Institute of Physiology and Pathophysiology, Johannes Gutenberg-University Mainz, 55099 Mainz, Germany

**Keywords:** photodynamic therapy, 5-aminolaevulinic acid, hyperthermia, infrared-A-radiation, rat sarcoma

## Abstract

The possibility of enhancing aminolaevulinic acid (ALA)-based photodynamic therapy (PDT) by simultaneous application of localised hyperthermia (HT) was evaluated. Treatments of rat DS-sarcomas included: (i) control, (ii) ALA administration (375 mg kg^−1^, i.p.), no illumination, (iii) ‘nonthermal’ illumination, (iv) ALA-PDT: that is, ALA administration, ‘nonthermal’ illumination, (v) localised HT, 43°C, 60 min (vi) ALA-PDT+HT: ALA administration with full spectrum irradiation resulting in ALA-PDT and HT. Tumour volume was monitored for 90 days or until a target volume (3.5 ml) was reached. No differences were seen between the first three groups, with all tumours reaching the target volume by 8–11 days. A total of 13 and 15% of tumours did not reach the target volume by day 90 following HT or ALA-PDT treatment, respectively. ALA-PDT+HT showed the greatest antitumour effect (*P*=0.0001), with 61% of the tumours not reaching the target volume. Viability and *in vitro* growth were also assessed in cells from tumours excised after treatment. ALA-PDT+HT reduced the fraction of viable tumour cells by 85%, and *in vitro* culture showed pronounced growth delay compared to control cells. These results demonstrate an enhanced antitumour effect upon ALA+HT, which appears to involve direct cell toxicity rather than solely vascular damage.

Photodynamic therapy (PDT) is a cancer treatment based on the photosensitisation of tumour cells and exposure to light with subsequent death of the malignant cells. One approach uses a natural porphyrin precursor, 5-aminolaevulinic acid (ALA), which enters the endogenous haem synthesis pathway. The final step in this pathway involves the incorporation of iron into protoporphyrin IX (PpIX) to produce haem. Due to the fact that many tumours exhibit abnormal levels of some of the enzymes involved in this pathway, a selective accumulation of PpIX – which is an efficient photosensitiser – can occur in tumour cells (Peng *et al*, 1997). PDT with ALA has been the focus of much clinical research and ALA is emerging as a promising agent for photodynamic treatment of lesions of the skin and internal hollow organs (Peng *et al*, 1997; Kelty *et al*, 2002). While PDT with ALA shows a number of advantages, such as reduced skin phototoxicity in comparison to other photosensitisers, its effect is usually superficial, a factor which may limit its clinical applicability. A number of investigations have been carried out to enhance the effects of ALA-PDT and have included the use of varying fluence rates and light fractionation (Messmann *et al*, 1995; de Bruijn *et al*, 1999; Iinuma *et al*, 1999), iron chelators (Curnow *et al*, 1998) and the use of ALA-esters (Peng *et al*, 1997). Other antitumour approaches include the combination of PDT with radiotherapy (Peng *et al*, 1997; Allman *et al*, 2000) or chemotherapy (Datta *et al*, 1997; Canti *et al*, 1998). Evidence is also available suggesting that the combination of PDT with hyperthermia (HT) may result in enhanced tumour response effects. In a number of *in vitro* studies, the combination of HT and PDT resulted in synergistic effects (Christensen *et al*, 1984; Mang and Dougherty, 1985; Rasch *et al*, 1996). Investigations carried out *in vivo* also showed promising effects (Henderson *et al*, 1985; Levendag *et al*, 1988; Kimel *et al*, 1992; Chen *et al*, 1996), although a thorough assessment of the optimum sequencing of the therapy components – especially in terms of a simultaneous application – has proven to be technically difficult. Waldow *et al* (1984) stated that although it may be desirable to carry out heat and light delivery simultaneously, they were prevented from doing this because of the practical limitations of using a microwave applicator for HT together with a laser for photosensitiser activation. From a clinical point of view, a therapy combination that could be applied in a single session would certainly be favourable. At the same time, consideration should also be given to the fact that the high costs of often complex laser systems probably represent the biggest obstacle to PDT gaining widespread clinical acceptance (Stringer, 1995). In the present study, an easily applicable technique has been used for simultaneous localised HT and ALA-based PDT using a single, nonlaser radiation source (Kelleher *et al*, 1999) in a rat tumour model. The experiments were designed to look at tumour growth *in vivo* and *in vitro* following treatment and to clearly elucidate the effects of the individual therapy components alone and in combination.

## MATERIALS AND METHODS

### Animals and tumours

The DS-sarcoma tumour growing in male Sprague–Dawley rats (Charles River Wiga, Germany; receiving a standard diet and water *ad libitum*, body weight at the time of tumour implantation: 180–210 g) was used in all experiments. Solid tumours grew subcutaneously following injection of DS-sarcoma ascites cells into the hind foot dorsum (0.4 ml; approx. 10^4^ cells *μ*l^−1^). This tumour was originally chemically induced in rats and arose in the auditory canal (Vaupel, 1974). Experimentation had previously been approved by the regional Ethics Committee, and was conducted according to the German Law for Animal Protection of 1987.

### Photosensitiser

5-Aminolaevulinic acid (Sigma, Germany) was dissolved in phosphate-buffered saline (PBS) at a concentration of 80 mg ml^−1^. This solution was slowly injected into a tail vein 3 h before illumination so that the animals received a dose of 375 mg kg^−1^ ALA (injection volume per rat ≈1 ml). After ALA injection, the animals were kept under subdued lighting conditions until treatment commenced. An equivalent volume of PBS was administered to control animals. The ALA dose was chosen on the basis of preliminary experiments, showing this dose to be the most effective in terms of tumour growth inhibition in combination with low toxicity. These experiments also showed this dose to be the lowest possible ALA concentration resulting in maximum ALA-induced fluorescence in the tumour investigated.

### Treatments

Once a tumour volume of 0.6–0.8 ml had been attained (6–8 days after tumour implantation), the animals were anaesthetised with sodium pentobarbital (40 mg kg^−1^ i.p., Narcoren®, Merial, Germany) and randomly allocated to the various experimental groups, the details of which are summarised in Table 1

**Table 1:** Description of parameters for the treatment groups

**Group no.**	**Treatment**	**ALA dose (mg kg^−1^, i.v.)**	**Drug – illumination interval (h)**	**Wavelength range (nm)**	**Fluence rate (mW cm^−2^)**	**Total energy dose (J cm^−2^)**	**Duration of illumination (min)**
I	Control	—	—	—	—	—	—
II	ALA-dark	375	—	—	—	—	—
III	Light alone	—	—	420–800	200	370	80
IV	ALA-PDT	375	3	420–800	200	370	80
V	HT	—	—	420–1400	600	1120	80
VI	ALA-PDT+HT	375	3	420–1400	600	1120	80

.

A radiation delivery system was used which has been described in detail previously (Vaupel *et al*, 1992; Kelleher *et al*, 1995, 1999). The light source in this system is a halogen lamp (24 V/150 W, type HLX64643, Osram, Germany) that emits over the spectral range from 420 to 1400 nm. The methods for activating the photosensitising drug or for inducing localised tumour HT were based on the same protocol. Since light fractionation and fluence rate can both affect the PDT tumour response, these parameters were carefully controlled, as described below. Additionally, since an ‘unconventional’ light fractionation was used for PDT in this study, further experiments were carried out that compared this fractionation with continuous light. Here, treatment with continuous light was carried out with the same drug and light dose as for the fractionated treatment (see Table 1, Group IV), except that the light was administered continuously over 30 min. As described above, the experimental groups were treated with light of different wavelengths. For groups III and IV, where ‘nonthermal’ illumination was required, a short wave-pass filter was inserted into the radiation path to remove wavelengths >800 nm, so that the infrared bands which would otherwise result in heating were removed. The full spectral range (420–1400 nm) was applied in those groups receiving HT (i.e., groups V and VI). Another feature of the light delivery system is a water-filter that is built in as a closed cuvette (Hydrosun, Germany) and is responsible for two strong, distinct absorption bands at 944 and 1180 nm which, when unfiltered are absorbed by the most superficial skin layers and can lead to painful sensations and exsiccosis. The water-filtered infrared component allows (at the same radiant flux density on a given surface) for longer exposure times and higher heat doses. Therapeutically relevant heating of tissue layers at a depth of up to 1.2 cm has been found to be possible with this system and the pattern of temperature gradients achieved has been described (Vaupel *et al*, 1992; Kelleher *et al*, 1995). The application of HT in this study (groups V and VI) involved the additional use of a feedback control system, where the temperature in the tumour centre was measured with needle-type thermocouples (φ 250 *μ*m; type 2ABAc, Philips, Germany) and the tumours heated at a rate of 0.4°C min^−1^ to a set temperature of 43°C (tumour centre) using the radiation source. Following a 20 min heat-up phase, the tumour temperature was maintained at this set level for 60 min, by continuous regulation of the source that switched on and off intermittently (total illumination time=80 min). This resulted in an average effective total energy dose of 1120 J cm^−2^ over the treatment period as measured in pilot studies. These pilot studies, in which HT was applied were also used to obtain information concerning the pattern of irradiation with the light delivery system used. An evaluation of these studies showed that, on average, tumours of the volume used in the present study were irradiated for a period of 1.3 s followed by a nonillumination period of 2 s, with this pattern continuing over the 80 min illumination period. This pattern was subsequently used for the groups requiring ‘nonthermal’ illumination so that the light delivered in the range from 420 to 800 nm was equal for all the illuminated groups, with a fluence rate of 200 mW cm^−2^ over this wavelength range for all treated groups. Additionally, the light hyperfractionation schedule was the same for all groups receiving irradiation. Light dosimetry was performed using a calibrated radiometer/photometer (IL1400A, International Light, USA). Irradiation was applied solely to the tumour, the remainder of the animal being shielded by aluminium foil.

On considering the treatment data outlined in Table 1, the fluence rate and light dose administered may appear high in comparison to that conventionally used during PDT. It should, however, be remembered that these parameters were not selected solely with PDT treatment in mind, but arose from the energy doses necessary to induce 43°C HT and the properties of the light source used. Lower fluence rates, at least for the ALA-PDT group, would have made a direct comparison between the different treatment groups inappropriate since identical fluence rates in the different groups for the wavelength ranges used would not have been applied.

### Assessment of tumour response *in vivo*

Prior to and following treatment, the three orthogonal diameters (*d*) of the tumours were measured on a daily basis and the tumour volume (*V*) was determined using the ellipsoid approximation *V*=*π*/6 × *d*_1_ × *d*_2_ × *d*_3_. In accordance with the guidelines of the UKCCCR (Workman *et al*, 1998), a target volume of 3.5 ml (selected so that the tumour burden by the end of the study never exceeded 1% of the animal's body weight) was used as the end point for the tumour growth study rather than survival. Where the tumour volume did not reach the target volume, the animals were monitored for up to 90 days.

### Assessment of tumour response *in vitro*

In a second series of experiments, tumours were treated *in vivo* and thereafter excised under sterile conditions, minced using a scalpel blade, and pressed through a cell dissociation sieve (380 *μ*m pore size, Sigma) using a glass pestle. A single-cell suspension was then prepared by incubating the tissue clumps in 30 ml culture medium (RPMI1640 (Sigma), supplemented with 10% bovine calf serum (HyClone, USA)) with 2 ml of an enzyme mixture containing 0.4 mg ml^−1^ DNAase (Roche, Germany) and 0.05 mg ml^−1^ collagenase (Sigma) for 20 min at room temperature, after which the cells were dispersed by repeated passage through a 22 gauge needle. Finally, the cells were washed, centrifuged and resuspended in culture medium (RPMI supplemented with calf serum as above). The cell number was then assessed in a haemocytometer, and cell viability determined by trypan blue exclusion (tumour cells from this cell line can be easily identified due to their characteristically large size). The cells were then decanted into tissue culture flasks at an initial concentration of 50 000 vital cells per millilitre growth medium, where they grew as a suspension in a humidified atmosphere of 5% CO_2_ in air for up to 6 days in the dark. The number of vital tumour cells was re-evaluated on a daily basis in a haemocytometer.

### Determination of ALA-induced fluorescence by flow cytometry

In order to assess differences in PpIX accumulation between tumour tissue and normal tissues, tumour-bearing animals were injected with ALA or PBS as described above. At 3 h after injection, tumours and normal tissues (liver, kidney, spleen) were excised and mechanically dispersed to obtain a single-cell suspension. Flow cytometric measurements were performed using an EPICS XL-MCL system (Beckman-Coulter, USA) to determine ALA-induced fluorescence. For this, the cells were illuminated by an argon laser (wavelength 488 nm) and the mean fluorescence was assessed in the wavelength range 605–635 nm. Tissues of animals receiving PBS served as controls and ratios between ALA-treated and control tissues were calculated.

### Statistical analysis

*In vivo* tumour growth characteristics were calculated using Kaplan–Meier statistics and the significance of the differences between the various groups was assessed using a log-rank analysis. All other analyses were carried out using the Wilcoxon test for unpaired samples. The significance level in all cases was set at *α*=5%.

## RESULTS

**Figure 1:**
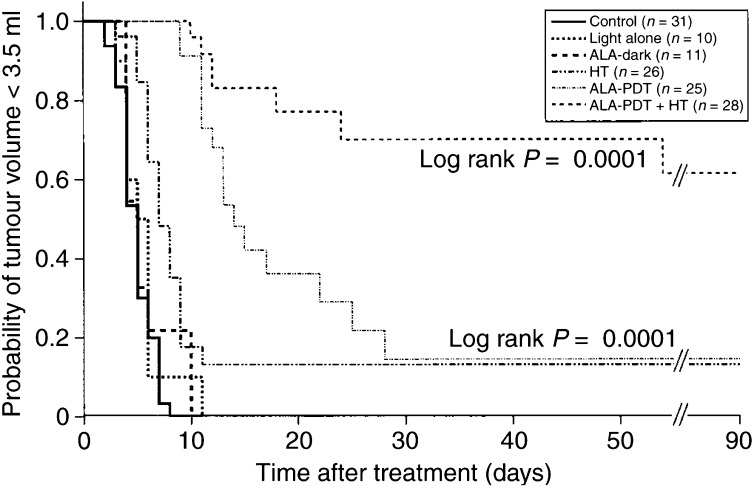
Kaplan–Meier analysis showing the probability of tumour volume being less than 3.5 ml as a function of time after control (sham-treatment), light alone, ALA-dark, HT (43°C, 60 min), ALA-PDT or combined ALA-PDT+HT treatment. *n*=number of animals treated.

Following treatment, animals from all groups were found to recover rapidly, with no side effects on general well-being or phototoxic effects on normal skin being recorded. Tumour size was assessed either until a tumour volume of 3.5 ml was attained (at which point animals were humanely killed) or up until 90 days after treatment. The probability of the tumour volume being less than 3.5 ml at any time point during the study was calculated using a Kaplan–Meier analysis (Figure 1

). On comparing the control group with the ALA-dark or light alone groups, no significant differences were found, with all tumours reaching the target volume by approx. 10 days after treatment. Tumour treatment with either ALA-PDT or HT alone caused a significant increase in the probability of tumours not reaching the target volume compared to the control group (*P*=0.0001, in both cases). Although the probability of not reaching the tumour target volume 90 days after treatment was approx. 15% in the case of either ALA-PDT or HT treatment, the log-rank test showed the course of the curves from these two treatment groups to be significantly different (*P*=0.0006), with the ALA-PDT group demonstrating an enhanced tumour growth delay up to 30 days following treatment, as seen in the corresponding right-shift in the early phase of the Kaplan–Meier curve. Treatment of tumours with ALA-PDT+HT produced the most pronounced antitumour effect, with a probability of the tumour not reaching 3.5 ml by 90 days after treatment of 61%, an effect which was significantly greater than that seen in the control, HT and ALA-PDT treatment groups (*P*<0.0001, in all cases). Of the animals monitored till 90 days in the ALA-PDT+HT group, none showed any evidence of tumour presence on macroscopic examination. The combined treatment therefore produces an antitumour effect which appears to be more than additive in comparison to the two component treatments alone. It is important to note that the assessment of true synergism, as described by Steel and Peckham (1979), was not within the scope of this study since it would require dose–response curves for both the therapy components. During ALA-PDT, the mean tumour temperature rose from 36.2±0.2°C (*n*=25) prior to the treatment to 37.6±0.16°C (175 temperature measurements) during the treatment, with a peak temperature of 38.4±0.12 (*n*=25). These temperatures are within the normal physiological range for rat rectal temperatures and therefore an HT component in the ALA-PDT treatment is unlikely. Experiments comparing the effects of fractionated and continuous light delivery schedules in ALA-PDT treatment showed no significant difference on log-rank analysis, with the probability of the tumour not reaching the target volume of 14.5% (*n*=25) by 90 days with the fractionated light schedule and 15% (*n*=11) with the continuous light schedule. Thus an influence of light fractionation on the PDT effect in this setting can be ruled out.

**Figure 2:**
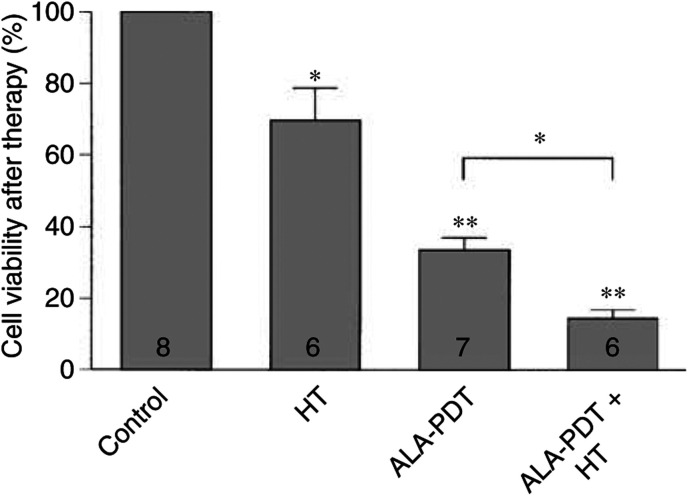
Cell viability following treatment with HT (43°C, 60 min), ALA-PDT or combined ALA-PDT+HT treatment, expressed relative to the values found in control (sham-treated) tumours. The values are means ±s.e.m., and the number of tumours investigated is indicated at the base of the columns. The significance levels indicate differences between control and treatment groups or between treatment groups as indicated. ^**^P<0.005, ^*^P<0.05.

**Figure 3:**
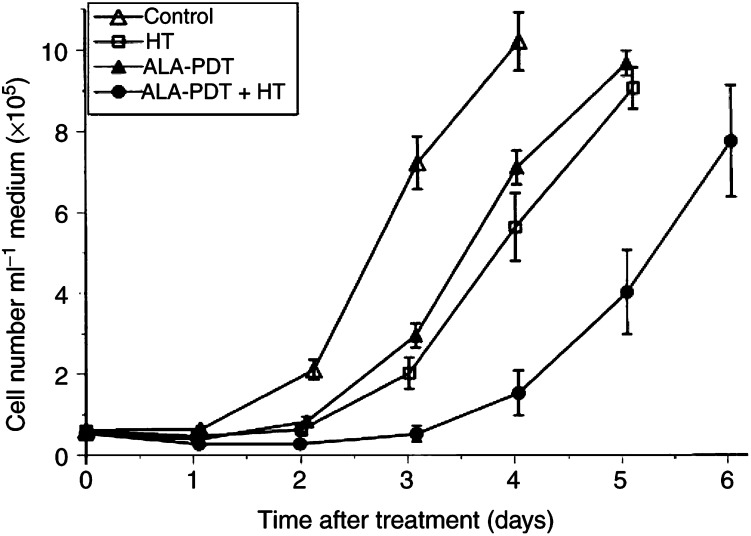
Tumour cell number as a function of time following *in vivo* treatment. A single-cell suspension for *in vitro* culture was prepared immediately after treatment from tumours undergoing sham-treatment (control), HT (43°C, 60 min), ALA-PDT or combined ALA-PDT+HT treatment. Each data point represents means±s.e.m. obtained with cells excised from a minimum of six tumours.

In further experiments in which tumours were excised immediately after treatment, the degree of cell viability was assessed (Figure 2

). When compared to control tumours, HT or ALA-PDT treatments significantly reduced the fraction of surviving vital cells, by 31 and 67%, respectively, with an even greater effect being seen following ALA-PDT+HT, where 86% of the tumour cells were no longer viable (*P*<0.005). Subsequently, the growth of viable cells from tumours of the different treatment groups was monitored *in vitro* (Figure 3

). In cells from control tumours, a doubling time of 0.75 days was calculated from the growth curves over the exponential growth phase, which was prolonged to 0.86, 0.93 and 1.24 days for cells from ALA-PDT, HT and ALA-PDT+HT treated tumours, respectively. When the numbers of cells on day 4 following treatment and excision were compared, all treatment groups had a significantly lower number of cells than the control group, and, in the case of the combined treatment, the cell number was significantly lower than with either of the component treatments (*P*<0.01, in all cases).

**Figure 4:**
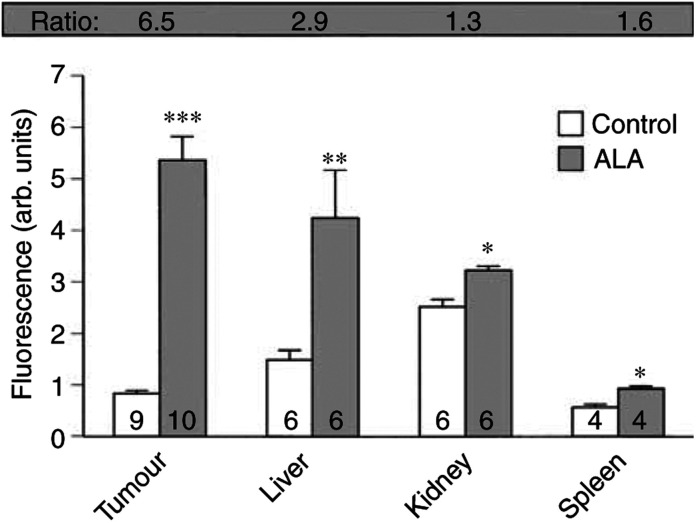
Mean fluorescence (at 605–635 nm) in tumour and normal tissues as determined by flow cytometry. Tumour-bearing animals were injected with ALA (375 mg kg^−1^, dissolved in PBS 80 mg ml^−1^) or equivalent volumes of PBS. After 3 h, single-cell suspensions from tissues of interest were obtained and ALA-induced fluorescence was measured by flow cytometry. Ratios between ALA-treated and control tissues were calculated and are shown in the shaded bar. The values shown are means±s.e.m. with the number of samples at the bases of the columns. Significance levels from differences between tissues obtained from ALA-treated and control animals; ^***^P<0.001, ^**^P<0.01, ^*^P<0.05.

Results from the assessment of ALA-induced fluorescence are shown in Figure 4

. Tissues were excised 3 h after ALA administration and the extent of ALA-induced fluorescence was assessed. Control tissues were obtained from animals treated with PBS so that treatment/control ratios for each tissue type could be determined. The greatest accumulation of fluorescence was found in tumour cells, where more than six times the amount of fluorescence was measured compared to control tumour tissue. Although the method used cannot exclude the contribution of other cell types within the tumour mass and will additionally not register any fluorescent products accumulating in the tumour interstitium, it would appear that DS-sarcoma cells are capable of accumulating ALA and converting it into fluorescent substances to a greater extent than the normal tissues studied. One of the fluorescent products of ALA metabolism is PpIX, which is thought to be predominantly responsible for the ALA-induced photodynamic effect. Ideally, in order to show selectivity of ALA uptake and conversion to fluorescent products in tumour tissue, a comparison of the uptake in normal skin and tumour in ALA-treated animals would have been appropriate. However, the mechanical dispersion of skin to obtain the required single-cell suspension was not possible, and an enzymatic dispersion would have required a moderate incubation period, thus not allowing determination of fluorescence 3 h after ALA administration. Here, a recent study by Perotti *et al* (2002) is noteworthy, which examined porphyrin synthesis using spectrofluorometry following ALA administration and found a similar tumour to liver ratio (1.3) to that reported here (1.1). At the same time, they obtained a tumour to skin ratio of 2.94. Assuming that the skin uptakes in the two studies are comparable, the results would appear to suggest a selective uptake of and conversion of ALA by the DS-sarcoma used in the present study, which may reflect tumour-specific differences in the haem metabolic pathways and/or differences in ALA uptake.

## DISCUSSION

Although HT alone is not considered an adequate therapy form for the curative treatment of human tumours, a number of clinical studies have demonstrated a clear benefit when HT is combined with other therapy modalities, in particular radiotherapy (Overgaard *et al*, 1996; Vernon *et al*, 1996; Prosnitz *et al*, 1999; van der Zee *et al*, 2000). A number of experimental studies, both *in vitro* and *in vivo* have also suggested a possible role for HT in combination with PDT, a therapy form that is currently being applied to a number of malignant and nonmalignant conditions (Ackroyd *et al*, 2002). In *in vitro* studies, the photodynamic process was found to be inherently temperature dependent (Gottfried and Kimel, 1991), and in a range of normal and tumour cell systems, the combination of HT and PDT was invariably found to be additive, if not synergistic, in terms of its cytotoxic effect (Christensen *et al*, 1984; Mang and Dougherty, 1985; Chen *et al*, 1996; Rasch *et al*, 1996). Most of these studies focused on identifying an optimal time interval between the two therapy components, and generally found that HT followed by PDT led to additive effects, whereas PDT followed closely by HT produced greater than additive effects, suggesting that in this sequence, the two modalities interact in a manner to induce cytotoxicity which is greater than the sum of the individual treatments. A similar pattern was found in *in vivo* experimental studies. Henderson *et al* (1985) examined the effects of photofrin-based PDT using a laser for photoactivation in combination with microwave-induced HT in RIF-mouse tumours. In these animals, PDT followed by HT showed the greatest inhibition of *in vivo* tumour growth and reduction of *in vitro* clonogenicity of cells taken from these tumours after treatment, which was seen to be potentiated when compared to the individual treatments. Other groups reported similar results, and explained that due to practical limitations (e.g., the use of a laser for photoactivation together with the use of a microwave to induce heating), simultaneous PDT and HT could not be carried out, even though this may be desirable when considering application in the clinical setting (Waldow *et al*, 1984, 1985; Levendag *et al*, 1988; Chen *et al*, 1996). In a study using a very elaborate technical set-up involving both an argon-dye laser (for photoactivation) and a neodymium : yttrium-aluminium-garnet laser (for heat deposition), a simultaneous application could be carried out and an enhanced tumour cure seen (Mang, 1990).

The issue of HT in PDT treatment has been frequently discussed in the past. The conclusions drawn from many ‘PDT’ studies, in which high fluence rates were often used, were confounded because of tissue heating during therapy. Kinsey *et al* (1983) mentioned that many reports attributing cell killing solely to PDT are probably only partially correct, and that in many instances, the tissue heating induced will have contributed to the tumoricidal effect. An exact determination of the role of HT in PDT studies is difficult due to the fact that the rise in temperature during PDT is dependent on a number of variable parameters, including optical absorption and light scattering, exposure time, tissue properties, thermal conduction and tumour perfusion (Svaasand *et al*, 1983). One study – in which attempts were made to quantify heating in hamster tumours during PDT – documented temperatures of over 43°C in tumours and concluded that an unrecognised overlap of the effects of PDT and HT might complicate the interpretation of studies on the mechanisms underlying PDT (Leunig *et al*, 1994). Certainly, uncontrolled and unmonitored tissue heating may not be a reasonable option for enhancing the PDT effect since there appears to be a narrow temperature window for this enhancement. Waldow *et al* (1984) reported the absence of any tumour control enhancement at ‘sublethal’ temperatures (⩽39.5°C), but an enhancement at temperatures of between 40.5 and 44.5°C. Rasch *et al* (1996) found that the degree of synergism depended greatly on the temperature reached, with effects at 43°C being greater than at 49°C. In a colon carcinoma model, the effect of ALA-PDT was seen to be enhanced by HT at 40.5°C, which was chosen to mimic temperature elevations frequently seen during ALA-PDT in clinical studies. This combined treatment led to a slower tumour growth (monitored for 14 days) and greater tumour necrosis than that seen with the treatment components alone.

The aim of the present study was to use an easily applicable single, nonlaser radiation source enabling a simultaneous combination of HT and ALA-PDT, whereby the HT component was regulated to maintain a clearly defined and controlled pattern of tumour heating set at 43°C. While the irradiation source used has been previously described, this study has now identified the effects of HT and PDT alone, showing that the effects of the combined therapy were considerably greater than the effects of the sum of the individual components, which may indicate a synergistic interaction. The combined therapy resulted in complete tumour cure in over 60% of the tumours treated (assessed by macroscopic examination for up to 90 days following treatment), which was considerably greater than that achieved in a mouse colon carcinoma model with a lower temperature of 40.5°C, where only a growth delay was seen (Orenstein *et al*, 1999). As far as the applicability of this combined treatment in the clinical setting is concerned, the technical features of the irradiation source used would allow treatment of superficial lesions, including cutaneous or subcutaneous metastases and accessible mucosal tumours. Delivery of light to more inaccessible regions can also be envisaged with the use of fibre optic technology. Further experiments – in which tumour cell viability and growth *in vitro* were assessed following *in vivo* treatment – were designed to provide an indication of possible mechanisms underlying the combined treatment effects. Both HT and PDT are treatment modalities that can greatly influence the tumour vasculature and thus, tumour blood flow. In the literature, HT has been reported to have distinctly heterogeneous effects on tumour blood flow, whereby this variability may be at least partially explained by the use of different heating protocols in tumours with different histologies. In the DS-sarcoma used in the present study, HT has previously been shown to have a bimodal effect on tumour blood flow with an initial increase in perfusion of up to 20% being followed by a decrease down to 25% of pretreatment values (Kelleher *et al*, 1995). Microvascular damage, which in some instances can even result in blood flow stasis, has frequently been seen following PDT (Reed *et al*, 1988; Fingar *et al*, 2000; Dolmans *et al*, 2002). Investigations of the effects of ALA-PDT on the microcirculation have shown decreases in perfusion in a number of experimental and human tumours (Roberts *et al*, 1994; Svanberg *et al*, 1996; Herman *et al*, 1999) and in normal tissue (Leveckis *et al*, 1995). However, in some tumour models, a lack of significant changes in blood flow has also been reported (Herman *et al*, 1999). In light of these possible vascular effects of both HT and ALA-PDT, tumours were excised directly after treatment and their *in vitro* growth behaviour monitored so that the extent of direct tumour cell damage by each of the treatments could be assessed. Theoretically, were the effects of the single or combined treatments purely due to vascular damage, then a decrease in cell viability as assessed immediately after treatment should not be seen. At the same time, the growth of tumour cells following excision should not be affected. In this study, however, both HT and ALA-PDT were seen to result in decreased cell viability after treatment. This effect was even more pronounced following ALA-PDT+HT and therefore suggests that vascular damage alone is not responsible for the tumour cure seen *in vivo*. Additionally, the fact that a growth delay was observed for the surviving cells suggested that these cells, while still being viable, were nevertheless affected by treatment. Despite the low percentage of tumour cells remaining viable after ALA-PDT+HT treatment, subsequent *in vitro* growth was seen with cells of all excised tumours, a finding that somewhat conflicts with the *in vivo* results. One reason for this may be that ALA-PDT+HT treatment, while not killing all tumour cells during treatment, may cause delayed cell death as a result of vascular damage. Alternately, the ALA-PDT+HT treatment may reduce the number of viable tumour cells to the extent that the host immune system is able to deal with them. Indeed, PDT treatment itself appears to be able to effectively trigger an immune response through the generation of tumour-sensitised immune cells (Korbelik and Dougherty, 1999). A similar effect has been suggested for HT, which can enhance the host antitumour immune response via the expression of heat-shock proteins (Ito *et al*, 2001). The immune system could therefore be stimulated by both components of the ALA-PDT+HT treatment. Thus, although direct tumour cell kill appears to play a major role in the ALA-PDT+HT effect, immunological and/or vascular effects may explain the differences seen between the *in vitro* and *in vivo* findings in this study. Other potential factors that might be involved in the pronounced potentiation of the antitumour effects seen are a concerted action of PDT and HT on certain proteins or supramolecular structures (Prinsze *et al*, 1991) or a conversion of PDT-induced repairable lesions to irreparable lesions upon HT treatment (Chen *et al*, 2001). Additionally, increased lactic acid formation during HT, as has already been reported for this tumour model (Kelleher *et al*, 1995), may also result in a lower pH in tumour tissue, and since acidosis has been found to enhance the efficacy of ALA-PDT *in vitro* (Wyld *et al*, 1998), such metabolic changes within the tumour tissue may play a role in the effects of ALA-PDT+HT.

In conclusion, a tumour therapy is described, which is based on ALA-PDT being carried out simultaneously with 43°C HT using a single, easily applied, nonlaser radiation source. This combined treatment was found to be more effective than the sum of its components, an effect which appears not to be based solely on vascular damage. Investigations into the mechanisms underlying the effects of ALA-PDT+HT are to be carried out to more clearly define the interaction of the therapy components.
